# Personal

**Published:** 1904-08

**Authors:** 


### PERSONAL.

Dr. Thomas D. Davis, of Pittsburg, was elected president of the National Association of United States Pension Examining Surgeons, at its recent annual meeting at Atlantic City. The experience of Dr. Davis will be valuable to the association, and the next meeting will undoubtedly be a profitable one.

Dr. Harold N. Moyer, of Chicago, the accomplished editor of *Medicine,* was elected president of the American Medical Editors' Association at its recent meeting at Atlantic City.

Dr. Walter B. Chase, of Brooklyn, has been appointed visiting surgeon to the Bethany Deaconess's Home and Hospital in that city.

Dr. E. C. Dudley, of Chicago, president of the American Gynecological Society, will deliver the address in gynecology at the next meeting of the Canadian Medical Association, which opens at Vancouver, August 23, 1904.

Dr. John B. Murphy, of Chicago, was elected president of the Chicago Medical Society at its recent annual meeting. This is one of the best organised city medical societies in the country and in President Murphy has an ideal chief who will not fail to make further progress for the society along the lines of scientific inquiry and social improvement which it has already marked out for itself.

Dr. J. P. Runyan, of Little Rock, who has been for several years the secretary of the Arkansas State Medical Association, was elected to the presidency thereof, at the last annual meeting. This is a most fitting recognition of the meritorious services of Dr. Runyan.

Dr. W. S. Renner, of Buffalo, sailed July 26 on the Kronprinz Wilhelm, to spend his vacation in Europe. He was accompanied by Mrs. Renner.

Dr. F. Whitehill Hinkel, of Buffalo, has been appointed a delegate to represent the American Medical Association at the

Seventh International Congress of Otology to be held at Bordeaux, August 1-4, 1904, under the presidency of Dr. E. J. Moure, Bordeaux, editor of *Revue Hebdomaire de Laryngologie, d'Otologie et de Rhinologie.* Dr. Hinkel sailed July 21, on the Hamburg and will return the last week in August.

---

Dr. John B. Deaver, of Philadelphia, upon invitation delivered an address on Diseases of the prostate before the British Medical Association during its annual meeting at Oxford, July 26-29, 1904. Dr. Deaver's treatise on enlargement of the prostate is about to issue from the press of P. Blakiston's Son & Company.

---

Dr. G. A. Himmelsbach, of Buffalo, sailed for Europe, July 12, 1904, and will return early in September. He will make an extensive tour, including Great Britain and the continent.

---

Dr. Irving W. Potter, of Buffalo, has gone to Europe for a two months' holiday, having sailed July 12, 1904. His tour will include Germany, Italy, France, and England.

---

Dr. Charles Haase, of Buffalo, has been appointed junior physician at the Manhattan State Hospital, located at Central Islip.

---

Dr. Joseph Burke, of Buffalo, received the degree of bachelor of science at the commencement of Manhattan College, New York, June 21, 1904.

---

Dr. L. S. McMurtry, of Louisville, president of the American Medical Association, sailed on the Arabic, July 29, 1904, for a six weeks' tour in Europe. His itinerary includes Dublin, Belfast, Glasgow, Edinburgh, London, Paris, Brussells, and Antwerp. He is accompanied by his daughter, Miss Marie-Louise, and they will sail homeward by the Finland, from Antwerp, September 3.

---

Dr. Charles G. Stockton, of Buffalo, sailed for Europe, July 28, for a few weeks' tour in Great Britain and on the continent.

---

Dr. E. P. Hussey, of Buffalo, was elected president of the International Hahnemannian Association at its recent annual meeting, held at Rochester, N. Y.

